# Luxation erecta

**DOI:** 10.11604/pamj.2014.18.70.4538

**Published:** 2014-05-22

**Authors:** Issam Elouakili, Redouane Ouakrim, Younes Ouchrif, Mohammed Kharmaz, Farid Ismael, Mly Omar Lamrani, Ahmed El Bardouni, Mustapha Mahfoud, Mohamed Saleh Berrada, Mouradh El Yaacoubi

**Affiliations:** 1 Service de Traumatologie-Orthopédie, CHU Ibn Sina, Rabat, Maroc

**Keywords:** Luxation erecta, dislocation inferieur de l'épaule, résultat du traitement, dislocation erecta, anterior dislocation of the shoulder, treatment result

## Abstract

La luxation de l'épaule dans sa forme erecta est une variété rare elle représente 0.5% de toute les luxations de l'épaules. Peu de cas ont été signalés, et le résultat du traitement a été mal défini. Entre 2001 et 2010, 09 patients avec luxation erecta ont été évalue dans notre formation. Il s'agit de huit hommes et une femme d’âge moyen de 31,5 ans. L'épaule droite était luxée dans sept cas, et le mécanisme était direct chez deux patients. L'attitude de l'épaule a été typique dans tous les cas avec un membre supérieur en abduction forcée, bras en l'air et impossibilité de ramener le coude au corps. Aucun trouble vasculaire n'a été noté par contre une atteinte nerveuse a été retrouvé dans 5 cas avec une évolution simple. La radiographie de l'épaule de face a objectivé une luxation inférieure de la tête humérale et un axe diaphysaire au-dessus de l'horizontale dans toutes nos observations. Le traitement a consisté en une réduction sous anesthésie générale suivie d'un bandage type Dujarier pendant trois semaines. La luxation de l'épaule type erecta constitue la forme type de la luxation inférieure, son mécanisme est une chute sur le membre supérieur en grande abduction ou antépulsion. Le diagnostic clinique est facile, confirmé par la radiographie de l'épaule de face. Le pronostic fonctionnel à long terme est excellent.

## Introduction

La luxation de l'épaule, est définie par une perte de contact totale et permanente de la tête humérale avec la cavité glénoïde de la scapula se produisant au décours d'un traumatisme. C'est une des urgences en chirurgie orthopédique dans le sens où l'intervention doit intervenir rapidement. D'une part pour le risque de compression d'éléments nobles mais aussi pour l'avenir de l'articulation (déformation articulaire, instabilité, arthrose). Elle s'accompagne toujours d'une attitude vicieuse propre à chaque forme anatomopathologique. Nous nous proposons de rappeler la variété dite erecta, forme type des luxations inférieures. Notre série rapporte neuf observations colligées sur une période de 10 ans. L'objectif de notre travail est d'insister sur la rareté de ces luxations et de rappeler leurs particularités cliniques, thérapeutiques et évolutives.

## Méthodes

Nous avons réalisé une étude rétrospective colligée au service de traumatologie orthopédie du CHU de Rabat concernant neuf cas de luxation inférieure de l'épaule sur une période de 10 ans entre 2001 et 2010. Les patients se répartissaient en 8 hommes âgés de 18 à 45 ans et une femme âgée 38 ans. L'épaule droite était concernée sept fois. Les circonstances de survenue étaient dominées par: les accidents de la voie publique (5 cas); les accidents de sports (2 cas); et une agression (2 cas).

**Figure 1:**
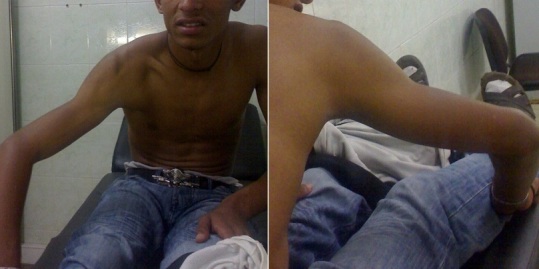
Présentation clinique d'une luxation erecta chez un jeune patient

Le mécanisme était direct chez deux patients par impact direct sur le moignon de l'épaule et indirect par chute sur la main ou le coude dans les autres cas. L'attitude de l'épaule était typique chez tous les malades qui se sont présentés avec un membre supérieur en abduction forcée, bras en l'air avec une impossibilité de ramener le coude au corps (Figure 1). Aucun trouble vasculaire n'a été rapporté chez nos patients par contre chez 5 cas nous avons noté une atteinte nerveuse qui a régressé totalement chez tous les patients.

**Figure 2:**
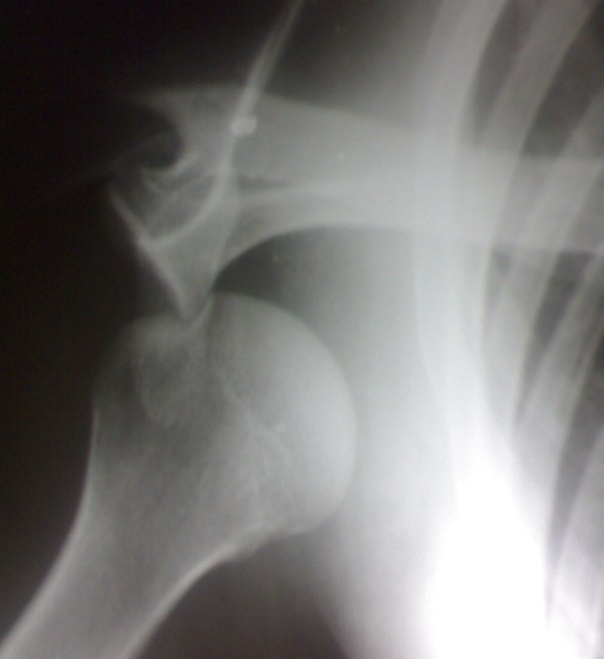
Radiographie de face de l'épaule objectivant une luxation erecta

**Figure 3:**
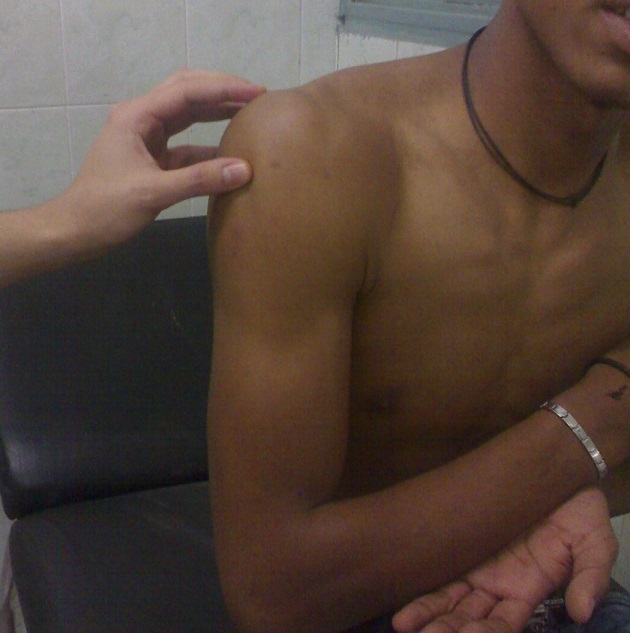
Images montrant la réduction de la luxation et la mise du membre en adduction

La variété erecta était confirmée par la radiographie de l'épaule de face qui objectivait la position sous-glénoïdienne de la tête humérale (Figure 2). La réduction était réalisée avec succès dans tous les cas. Elle consistait en une traction dans l'axe du membre avec légère abduction sous anesthésie générale (Figure 3), suivie d'une contention par bandage coude au corps, type Dujarier, pendant trois semaines. La rééducation précoce était préconisée chez tous les patients.

## Résultats

La durée moyenne de suivi était de 16 mois (8 mois-24 mois). L'élévation antérieure moyenne était de 150° (120°-180°). Nos résultats ont été évalués en se basant sur l'échelle de notation de l'UCLA [1] qui comprend une évaluation de la douleur, la fonction, l′amplitude des mouvements, la force, et la satisfaction du patient. Le score UCLA moyen dans notre série était de 30 points (extrêmes: 24-35 points). Six de nos malades avaient des résultats excellents, une réalisation parfaite des gestes de la vie quotidienne et une reprise totale de l'activité sportive. 2 malades ont gardé des douleurs résiduelles mais avec une amplitude qui dépasse les 140°. Un seul malade a gardé une antépulsion à 120° avec une bonne satisfaction des résultats du traitement. Nous n'avons noté aucun cas de récidive chez nos patients.

## Discussion

La luxation erecta représente une entité relativement rare, elle représentant seulement 0,5% de toutes les luxations de l′épaule [2, 3]. A notre connaissance il existe moins de 150 observations rapportées dans la littérature [2–5]. Davids et Talbott ont rapporté deux mécanismes de luxation erecta en 1990 [6]: un mécanisme direct par application de forces d'abduction violente sur un membre initialement en abduction, l'acromion servant de levier pour l'axe de l'humérus; et un mécanisme indirect suite à l'application d'une surcharge lourde sur un membre en abduction complète.

Gagey et al. ont décrit ce mécanisme, à propos de 32 luxations erecta expérimentales [7], par une simple élévation rotation externe du membre. Le terrain d'hyperlaxité ligamentaire a été rapporté dans plusieurs séries [8, 9]. Les accidents de la voie publique représentent l'étiologie principale suivi des accidents de sports [10, 11].

L′aspect clinique de la luxation erecta est pathognomonique avec le bras traumatisé en hyperabduction; le coude et la main soutenu par le membre controlatérale et le patient reste incapable d'abaisser le membre. L'examen clinique permet de palper la tête humérale au dessous de la glène scapulaire contre la paroi latérale de la cage thoracique [12, 13]. Bien que le diagnostic positif puisse être posé cliniquement, un bilan radiologique s'avère nécessaire pour confirmer la luxation et révéler les éventuelles lésions associées. La radiographie standard de face et un vrai profil axillaire permettent de montrer la tête humérale projetée sous le pôle inférieur de la glène [14]

L'étude expérimentale de Gagey et al. [7] ont décrit les différentes lésions anatomopathologiques de cette forme à travers les résultats de l'IRM de 24 luxations récidivantes. Selon cette étude, la lésion du ligament glénohuméral inférieur ainsi que celle du bourrelet adjacent était constante. La luxation erecta se produisait lorsque la déchirure du ligament glénohuméral inférieur était longitudinale. Pour que la luxation survienne expérimentalement, il fallait, dans sept cas sur huit, une désinsertion de la face profonde de la coiffe des rotateurs.

La luxation erecta doit être réduite en urgence sous anesthésie générale. Parmi les techniques de réductions les plus efficace rapporté dans la littérature et la technique de traction-contre traction [12, 13] qui consiste en une traction du bras dans l'axe du membre pendant que l'aide applique un contre appui sur le thorax. Le bras ensuite ramené en adduction et une immobilisation coude au corps est gardé pendant 3 semaine. Une radiographie post-réduction doit être faite afin de confirme la réussite de la réduction et de décelé d'éventuelle fracture iatrogène.

Les résultats de notre expérience rejoignent celle de la littérature concernant l'atteinte nerveuse [12, 13]. Nous avions noté une lésion nerveuse périphérique chez 5 de nos malade toute au dépend du nerfs axillaire; ces lésion ont régressé après réduction dans un intervalle de 1jour à 8mois. Aucune atteinte vasculaire n'a été rapportée dans notre série. Garcia et al. ont rapporté un cas de luxation erecta bilatérale compliqué d'une thrombose de l'artère axillaire imposant un traitement anticoagulant [15] et, dans la série de Mallon et al. [4] comportant 86 observations, ont été rapportées une atteinte du nerf axillaire dans 60% des cas et une atteinte de l'artère axillaire dans 3% des cas. La forme bilatérale de la luxation erecta a été rapportée par plusieurs auteurs [16, 17]. En conclusion il semble que les luxations erecta sont de bon pronostic contrairement à se qui a été rapporte par quelques auteurs [18].

## Conclusion

La luxation type erecta est une affection rare. Son diagnostic est posé cliniquement et confirmer par la radiographie standard. En raison du déplacement important de la tête humérale, les complications vasculo-nerveuses sont fréquentes. La triade réduction, bandage et rééducation précoce constitue le garant d'une bonne évolution. Une stabilisation chirurgicale peut être proposée pour les luxations récidivantes.
